# Ultra-Wideband Quad-Parallel Shunt-Diode Rectifier for Sub-6 GHz Wireless Power Transfer

**DOI:** 10.3390/mi16121417

**Published:** 2025-12-17

**Authors:** Sadık Zuhur

**Affiliations:** Department of Electricity and Energy, Igdir University, Igdir 76000, Türkiye; sadik.zuhur@igdir.edu.tr

**Keywords:** RF-DC conversion, Schottky diode, FR4, 5G, impedance matching

## Abstract

Wireless power transfer via RF/microwave rectifiers has emerged as a sustainable solution to the energy requirements of low-power devices. In this study, a novel four-parallel-shunt-diode ultra-wideband rectifier is proposed to enable wireless power transfer in the sub-6-GHz 5G bands. The proposed circuit maintains a power conversion efficiency (PCE) above 50% across the 1.6–5.1 GHz frequency range at 10 dBm input power and also achieves an efficiency above 50% at 3 GHz for input powers between 1 dBm and 16 dBm. Designed and fabricated on a low-cost FR4 substrate, the rectifier achieves a maximum power conversion efficiency of 76% at 2.9 GHz with a 10 dBm input power. Furthermore, a wideband impedance analysis is performed, taking into account the packaging parasitics of the HSMS-2860 diodes used in the study. Despite the use of a lossy substrate such as FR4, the proposed four-parallel-shunt-diode topology improves impedance stability and provides impedance matching over both a wide input-power range and a wide frequency band when compared with single- and double-diode structures reported in the literature.

## 1. Introduction

In recent years, radio frequency (RF) and microwave wireless power transfer (WPT) technology has emerged as a sustainable solution for meeting the energy demands of low-power electronic devices and has attracted significant attention from the academic community. WPT not only provides greater system flexibility but also has the potential to extend the lifetime of systems and reduce maintenance requirements by eliminating faults associated with wired connections [1,2].

Recent studies indicate that short-range WPT has been investigated at input power levels around 10 dBm for applications such as the Internet of Things (IoT), radio frequency identification (RFID) systems, and wireless sensor networks (WSNs) [3,4,5,6]. These studies aim to deliver sufficient energy to enable the operation of low-power electronic devices.

In WPT systems, rectifiers are key components that convert RF energy into direct current (DC) power. The effective and efficient operation of the rectifier directly affects the overall system performance. Therefore, the primary design objectives of a rectifier can be defined as achieving high power conversion efficiency (PCE), wide frequency bandwidth, broad input power dynamic range, compact size, and low cost.

5G technology offers new opportunities for WPT at higher frequencies. With the adoption of 5G frequency bands, the increasing proliferation of antennas and the capability to transfer high-density power are expected to positively impact the performance of WPT systems [7,8]. Consequently, numerous innovative rectifier designs and techniques have recently been proposed in the literature for 5G frequency bands [9,10,11]. However, while most of these studies focus on one or a few of the aforementioned design parameters, the present work considers all of them to propose an effective and efficient RF/microwave rectifier.

The proposed design was realized by connecting four shunt diodes in parallel. In the literature, shunt-diode-based rectifiers generally are employed single or dual-diode configurations [12,13,14] typically operate in one or two frequency bands or within a narrow bandwidth.

In [15], a dual-band rectifier based on an inverse Class-F architecture was proposed, achieving 64% efficiency at 2.4 GHz and 3.5 GHz through harmonic control. Another compact single-diode design [16] was achieved 80% efficiency at 2.45 GHz and 5.8 GHz. In [17], a rectifier employing two parallel shunt diodes was presented, where the admittances of two parallel transmission lines were optimized to be close to 50 Ω. Impedance matching at two frequency points enabled the dual-band design to be extended into a broadband rectifier operating over the 1.64–3.18 GHz range.

However, limiting the number of diodes in these structures prevents full compensation of the diode impedance variation with frequency, resulting in fluctuations in impedance stability across the operating band in WPT applications. Therefore, in this study, a design methodology based on the impedance model of the Schottky diodes was developed to achieve broadband impedance stability using four parallel shunt diodes.

The proposed ultra-wideband (UWB) rectifier provides significant innovations and contributions to the literature in the field of RF/microwave WPT. Firstly, the quad-parallel shunt-diode topology employed significantly enhances impedance stability and wideband performance compared to the single- and dual-diode structures commonly reported in the literature. This architecture enables the rectifier to maintain a power conversion efficiency (PCE) exceeding 50% across the entire 1.6–5.1 GHz band, enabling efficient RF-DC conversion in the sub-6 GHz 5G bands. The rectifier also exhibits a wide input power operating range, maintaining PCE values above 50% for input levels between 1 dBm and 16 dBm, which makes it highly tolerant to power fluctuations typically encountered in practical WPT scenarios. The design employs a low-cost FR4 substrate instead of high-performance dielectric materials such as RO4350B or Taconic TLY-5, which are frequently used in the literature. Additionally, its compact size of 19 × 27 mm^2^ facilitates seamless integration into applications such as IoT devices, RFID tags, and WSNs. In this article, the impedance behavior of the Schottky diodes is examined in Section 2. The proposed topology is analyzed in Section 3. The results obtained from simulations and experimental measurements are compared with related studies in the literature in Section 4. The key conclusions and recommendations are presented in the final section.

## 2. Schottky Diode Impedance Model and Design Approach

**Figure 1:**
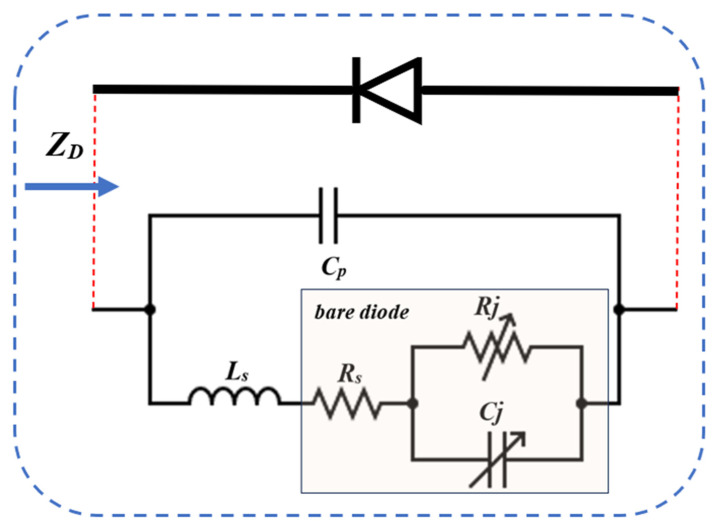
Small-signal equivalent circuit model of a Schottky diode, illustrating the intrinsic junction resistance and capacitance together with the series resistance, package parasitic inductance, and capacitance.

In this study, four Schottky diodes were employed; therefore, the impedance characteristics of the diodes were initially analyzed as part of the design procedure. Figure 1 illustrates the small-signal equivalent circuit model that represents the electrical behavior of a Schottky diode, including the parasitic components originating from the package. In this model, R_s_ denotes the series resistance of the intrinsic diode, R_j_ is the junction resistance, and C_j_ is the junction capacitance. The value of R_s_ remains essentially constant with respect to both frequency and input power, whereas R_j_ varies as a function of the diode bias current (I_b_), and C_j_ varies with the junction voltage (V_j_) [18].

The current–voltage characteristics of the diode are described by the Shockley equation, as given in Equation (1). The junction resistance R_j_ and junction capacitance C_j_ are expressed in Equations (2) and (3), respectively. These expressions constitute the basis for calculating the diode impedance and enable a more accurate analysis of the diode’s behavior under varying signal conditions.

(1) $$I_{b}\;=\;I_{s}(e^{\frac{{qV}_{a}}{nk_{B}T}}\;-\;1)$$

(2) $$R_{j}\;=\;\frac{8.331\;\times \;10^{-5}nT}{I_{s}\;+\;I_{b}}$$

(3) $$C_{j}\;=\;\frac{C_{j0}}{{\left(1\;-\;\frac{V_{a}}{V_{j}}\right)}^{m}}$$

Equation (1) defines n as the diode ideality factor, V_a_ as the applied voltage, q as the electron charge, T as the absolute temperature (in Kelvin), and k_B_ as the Boltzmann constant. The junction resistance R_j_, expressed in Equation (2), varies with both temperature and the reverse saturation current (I_s_). The junction capacitance C_j_, given in Equation (3), depends on the zero-bias junction capacitance (C_j0_), the junction voltage (V_j_), and the junction grading coefficient (m).

In RF rectifier applications, parasitic effects originating from the diode package have a significant impact on the input impedance (Z_in_) [19]. At high frequencies, these parasitics introduce leakage inductance (L_s_) and package capacitance (C_p_), which lead to impedance deviations and mismatch conditions that degrade overall rectifier performance. Therefore, the frequency-dependent parasitic elements must be incorporated into the intrinsic diode impedance model. Equation (4) represents the impedance Z_bD_(ω) of the bare diode, derived solely from its nonlinear intrinsic characteristics. When the package parasitics are included, the extended diode impedance is obtained as shown in Equation (5), yielding Z_D_(ω), which more accurately reflects the diode behavior in practical high-frequency rectifier designs [20].

In RF applications, parasitic effects originating from the diode package significantly influence the input impedance (Z_in_) of the rectifier [14]. These parasitics introduce series parasitic inductance (L_s_) and parasitic junction/package capacitance (C_p_) at high frequencies, leading to impedance mismatches that degrade the overall rectifier performance. Therefore, it is necessary to incorporate the frequency-dependent parasitic contributions into Equation (4), which represents the impedance Z_bD_(ω) of the bare diode including only its intrinsic nonlinear characteristics [20]. Under these conditions, Equation (5) expresses the extended diode impedance Z_D_(ω), where the packaging parasitics are included in the model.

(4) $$Z_{bD}\left(\omega \right)\;=\;R_{s}\;+\;\frac{R_{j}}{1+j\omega R_{j}C_{j}}$$

(5) $$Z_{D}\left(\omega \right)\;=\;{\left(\frac{1}{Z_{bD}+j\omega L_{s}}+j\omega C_{p}\right)}^{-1}$$

The model presented in Equation (5) characterizes the frequency-dependent impedance Z_D_(ω) and provides a mathematical representation of the actual nonlinear behavior of each diode used in the design. To achieve improved impedance stability and wideband operation, four Schottky diodes are connected in parallel in the proposed rectifier topology. Consequently, the total equivalent impedance Z_eq_ of the parallel diode network is obtained as given in Equation (6).

(6) $$Z_{eq}=\frac{Z_{D}}{4}$$

In the proposed four-parallel shunt diode configuration, impedance fluctuations caused by variations in frequency and input power are reduced to approximately one-fourth of those observed in single- or dual-diode structures. This reduction results in a significantly more stable input impedance across the operating band. Consequently, the parallel-diode approach provides a robust foundation for the impedance-matching strategy and the subsequent optimization procedures employed in the rectifier design.

## 3. Wide-Band Operation Analysis

**Figure 2:**
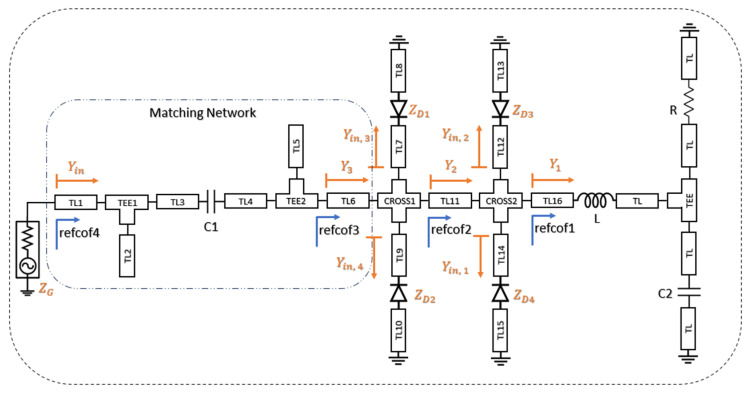
Architecture of the proposed ultra-wideband rectifier, employing four parallel shunt-connected diode branches together with the transmission-line network designed to stabilize the input impedance and enable broadband matching.

During the design process, the input impedance of the proposed rectifier was adjusted to approach 50 Ω. Four parallel shunt diodes were employed to compensate for the variations in diode impedance with respect to frequency and input power, and the proposed rectifier architecture is illustrated in Figure 2. This configuration enables broadband impedance matching by mitigating impedance fluctuations and nonlinear effects. Each branch includes transmission lines with a specific characteristic impedance (Z_0k_) and electrical length (θ_k_), while the shunt diodes exhibit impedances Z_D_(ω, P) that vary with frequency and input power. Based on these parameters, the load impedance in each branch can be calculated using Equation (7). Accordingly, the input impedance of each branch is expressed by Equation (8), and the corresponding input admittance is given in Equation (9).

(7) $$Z_{L,i}\;=\;f(Z_{D}(\omega ,P),{\;Z}_{0k},{\;\theta }_{k})$$

(8) $$Z_{in,i}=Z_{0i}\;\frac{Z_{L,i}+jZ_{0i}tan\theta_{i}}{Z_{0i}+jZ_{L,i}tan\theta_{i}}$$

(9) $$Y_{in,i}=\frac{1}{Z_{in,i}}$$

**Figure 3:**
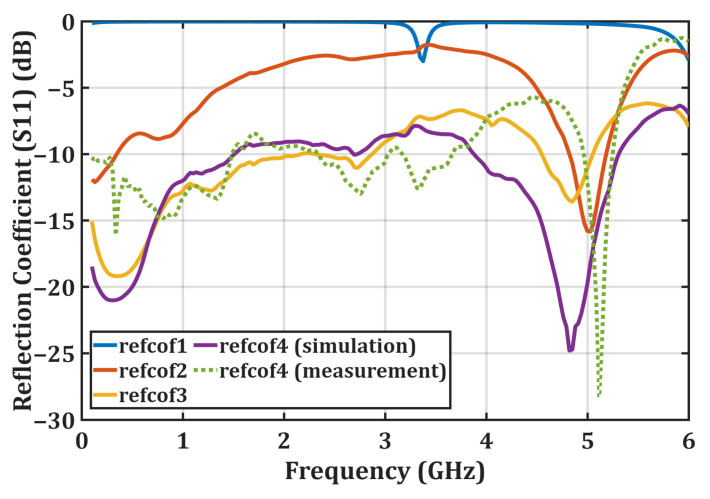
Simulated reflection coefficients (S11) for the design stages (refcof1–refcof4) and the measured S11 of the final configuration, illustrating the progressive improvement in impedance matching across the operating band.

**Figure 4:**
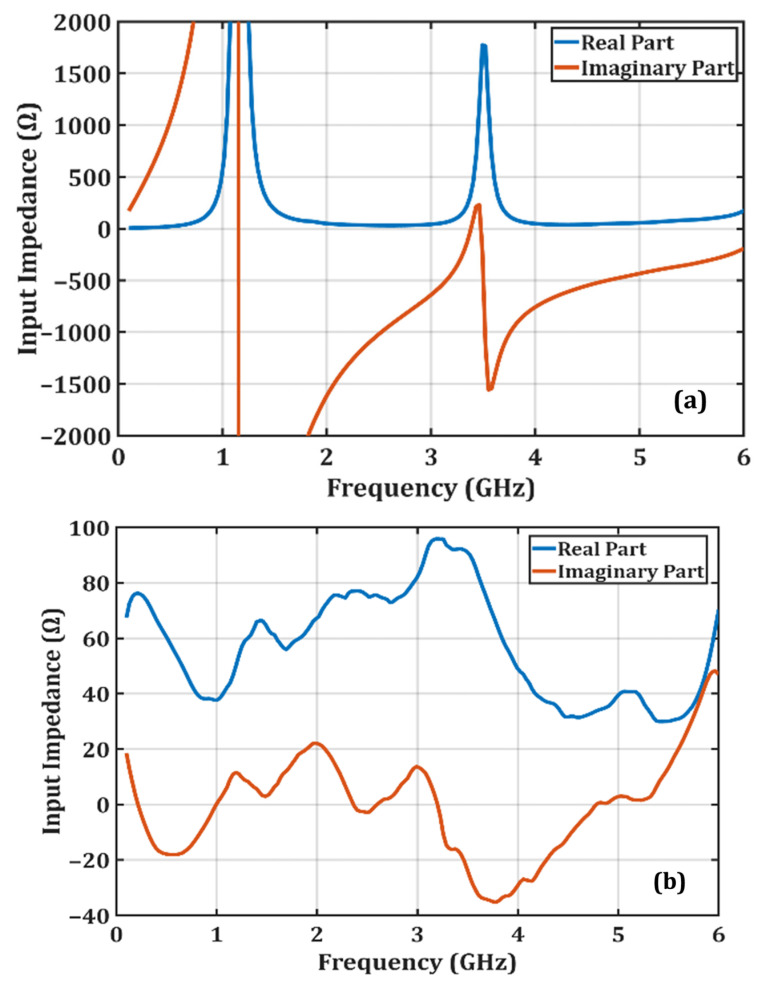
(**a**) Input impedance of the refcof1 configuration before the addition of the four shunt-diode structure and the matching network, showing large reactive deviations due to the absence of impedance compensation. (**b**) Input impedance of the final refcof4 configuration after incorporating the four shunt-diode structure and the matching network, demonstrating a significant convergence toward 50 Ω.

Figure 3 shows the variation in the reflection coefficient S_11_ in the simulation environment during the design process. Here, the refcof1 configuration represents the condition without the parallel branches and without the matching network. In this case, the RF choke used to prevent the high-frequency RF signal from propagating toward the DC output is selected with a large inductance value, resulting in a very high impedance at high frequencies. This behavior is observed in Figure 4a, which shows the input impedance before impedance matching; in this graph, the reactive component of the input impedance takes excessively large values. Consequently, the admittance Y_1_ approaches zero, and the circuit behaves like an open circuit.

In the refcof2 configuration shown in Figure 2, the reflection coefficient S_11_ was examined by adding branches 1 and 2 to the design. The refcof2 curve is lower than the refcof1 curve, indicating a partial impedance match. In this case, the input admittance is given by Y_2_ = Y_in,1_ + Y_in,2_. In the refcof3 configuration, all four proposed parallel branches were added to the design without a matching network, resulting in a quadruple parallel shunt-diode topology. At this stage, the input admittance becomes Y_3_ = Y_in,1_ + Y_in,2_ + Y_in,3_ + Y_in,4_, reducing the effective diode impedance to approximately Z_D_/4. This effect is clearly visible in Figure 2, and the impedance matching is largely achieved. However, an additional important point is the optimization of the transmission lines used for interconnection (TL9, TL10, TL11, TL12, TL13, TL14, TL15, TL16, TL17, TL18) during the additions. The optimization of these transmission lines also contributes to the broadband matching achieved. This optimization was performed using a gradient-based method with the Keysight ADS Harmonic Balance analyzer, with the primary goal of maximizing the rectifier output voltage.

Finally, the refcof4 configuration in Figure 2 represents the final state of the proposed rectifier. In this configuration, the optimization process was completed by adding an impedance matching network to the four-arm parallel diode structure. Figure 3 shows that the inclusion of the matching network further reduces S_11_ at high frequencies. This occurs because the matching network suppresses the capacitive effects and back-reflected components exhibited by the Schottky diodes at high frequencies. Moreover, the input impedance curve obtained from the simulation results after matching is completed, shown in Figure 4, indicates that impedance matching has been largely achieved. Figure 3 presents the curves obtained from both simulation and experimental measurements, and it is observed that these curves exhibit close agreement. However, due to the limited operating range of the Schottky diodes used and the frequency-dependent variations in the dielectric constant and loss tangent of the FR4 substrate, discrepancies between the simulation and measurement results were observed at the low and high ends of the operating band.

## 4. Results and Discussion

In this study, the proposed quad-parallel shunt-diode rectifier was developed and tested for UWB operation between 1.6 GHz and 5.1 GHz. Simulations were carried out in the Keysight ADS (Keysight Technologies, Santa Rosa, CA, USA) environment during the rectifier design process. Broadcom (Avago, San Jose, CA, USA) HSMS-2860 Schottky diodes, whose fundamental parameters are given in Table 1 and which can operate between 915 MHz and 5.8 GHz, were used in the design. These diodes were selected due to their low junction capacitance and their frequent use in wideband WPT applications. A cost-effective 1.6 mm thick FR4 substrate, widely used in industry, with a relative permittivity (ε_r_) of 4.6 and a loss tangent (tan δ) of 0.01 was used in both the simulation and fabrication processes.

**Figure 5:**
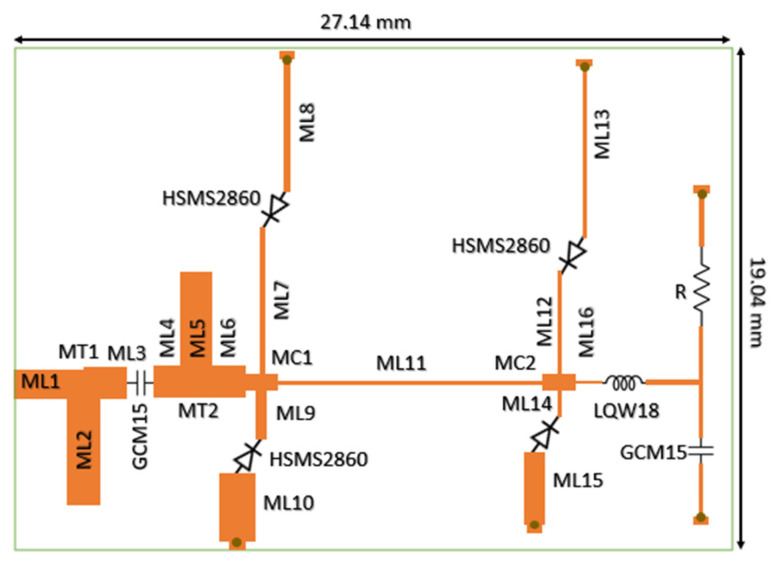
Layout of the fabricated ultra-wideband rectifier prototype, showing the optimized transmission-line structure and the four parallel shunt-diode network used for wideband impedance matching.

Figure 5 shows the layout of the prototype of the proposed rectifier fabricated for verification. During the design phase, the transmission lines were optimized to increase the power conversion efficiency (PCE), and the dimensions of the optimized transmission lines are given in Table 2. Table 3 presents the dimensions of the components that interconnect the transmission lines. A Murata 270 nH series inductor (LQW18ANR27G00, Murata Manufacturing Co., Ltd., Kyoto, Japan) and a 220 pF shunt capacitor (GCM1555C1H221JA16, Murata Manufacturing Co., Ltd., Kyoto, Japan) were used for filtering purposes. The rectifier, whose design process was completed in the simulation environment, was fabricated with a compact size of 19.04 mm × 27.14 mm, and the testing phase was initiated.

**Figure 6:**
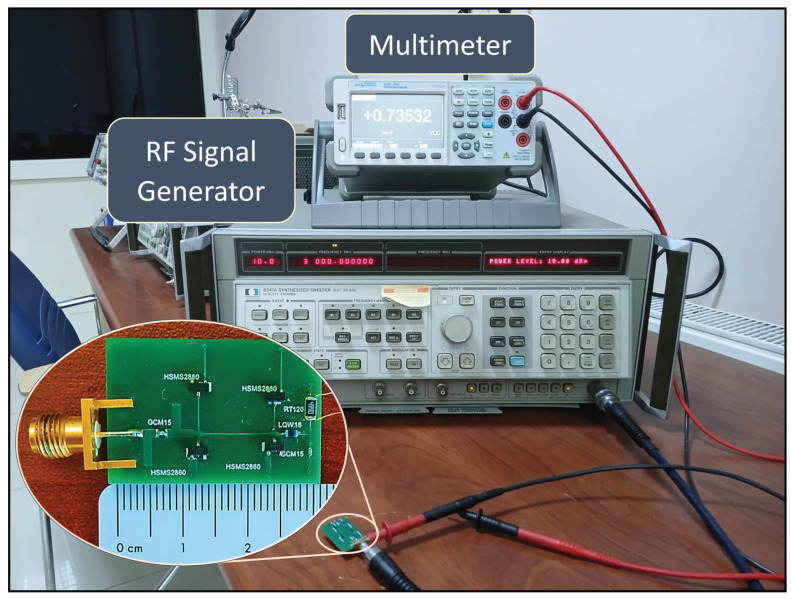
Photograph of the fabricated ultra-wideband rectifier prototype and the experimental measurement setup used for RF signal generation and DC output evaluation.

Figure 6 shows a photograph of the equipment and experimental setup used to test the UWB rectifier. The fabricated rectifier was tested using an HP8341 RF signal generator (Agilent Technologies, Santa Clara, CA, USA) and an AAtech digital multimeter (NETES, Istanbul, Türkiye). Additionally, the S_11_ measurement data shown in Figure 2 was obtained using a PicoVNA vector network analyzer (Pico VNA108; Pico Technology, Eaton Socon, Cambridgeshire, UK). The PCE calculation was performed based on the DC output voltage (V_out_) values measured at the output using Equation (10),

(10) $$PCE=\frac{P_{out}}{P_{in}}\;100\%=\frac{V_{DC}^{2}}{R_{L}\;P_{in}}\;100\%$$

where P_out_ represents the output power, P_in_ represents the input power, and R_L_ represents the load resistance of 499 Ω.

**Figure 7:**
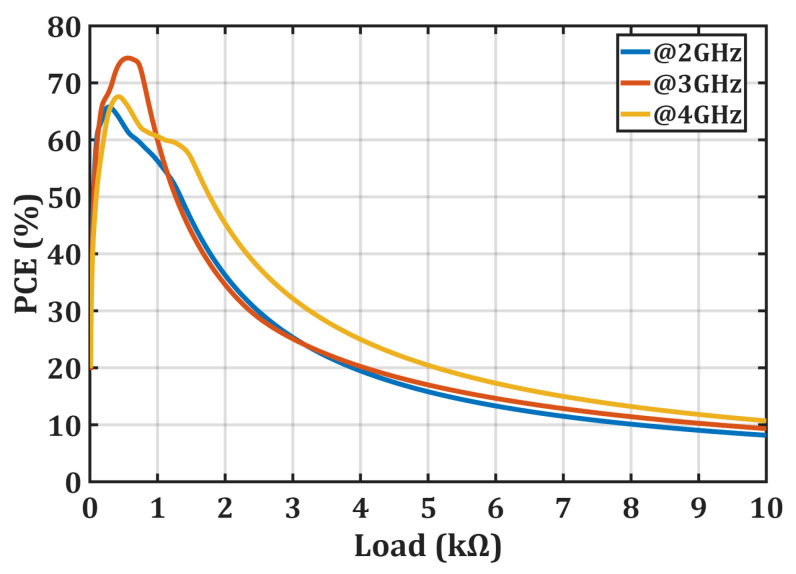
Simulated power conversion efficiency (PCE) of the proposed rectifier as a function of load resistance at different operating frequencies, demonstrating the load adaptability of the four-parallel diode structure.

Experimental evaluation was performed using a fixed load resistance of 499 Ω. However, the behavior of the rectifier under different load values was also investigated in simulation environment. Figure 7 shows the variation in PCE depending on the load resistance at different operating frequencies. The simulation results indicate that the rectifier maintains an efficiency of 50% or higher for load resistance values ranging from 60 Ω to 1.26 kΩ at operating frequencies of 2 GHz, 3 GHz, and 4 GHz.

**Figure 8:**
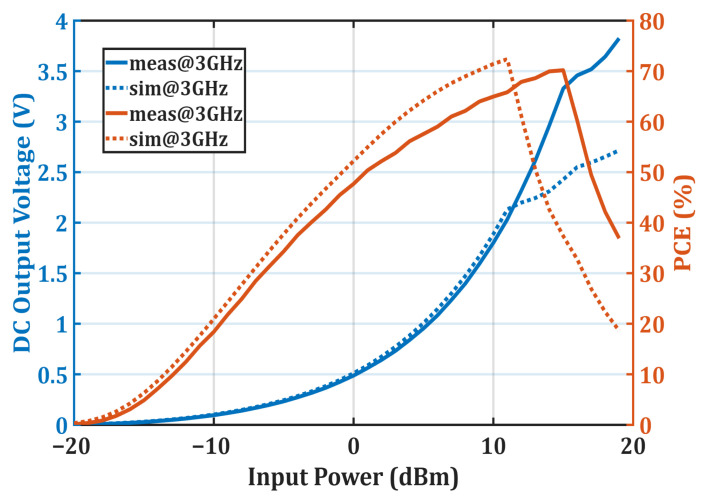
Measured output voltage and RF–DC conversion efficiency of the proposed rectifier as a function of input power at 3 GHz, highlighting the nonlinear diode behavior and the consistency between simulation and experimental results.

Figure 8 shows the V_out_ and calculated PCE values measured at 3 GHz as a function of input power, demonstrating the nonlinear behavior of the diodes with respect to input power. This graph indicates that the simulation and measurement results are highly consistent, and that PCE increases with rising input power up to 15 dBm. The proposed rectifier is observed to be highly effective against input power fluctuations, maintaining a PCE above 50% over the 1 dBm to 16 dBm input power range. At 15 dBm input power and 3 GHz, the measured V_out_ reached 3.5 V, while the PCE reached 70%. Beyond this input power level, the diodes entered saturation, resulting in a slower increase in V_out_ and a rapid decrease in PCE. According to the measurement results, the diode saturation point occurred at a higher input power value than in the simulations. This difference is primarily due to the diode model used in the simulation having a lower breakdown voltage (V_br_) compared to the actual diode.

**Figure 9:**
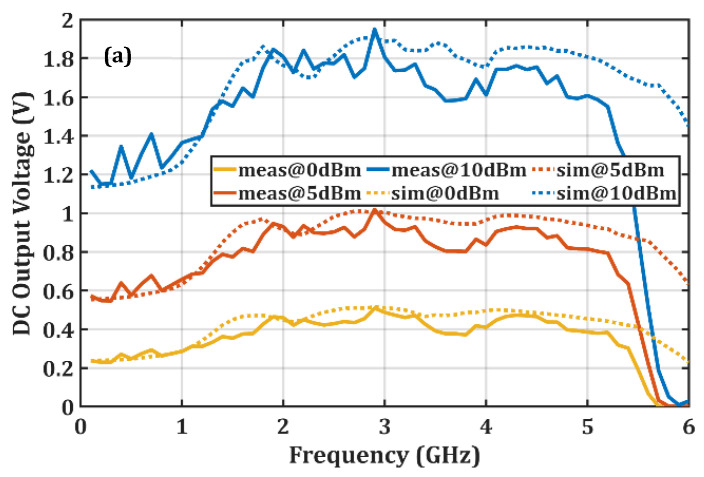

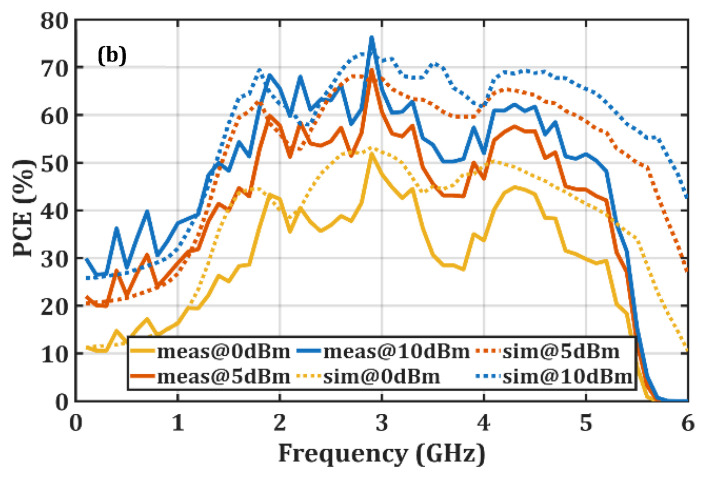
(**a**) Measured and simulated output voltage and (**b**) RF–DC conversion efficiency of the proposed rectifier as a function of frequency at 0 dBm, 5 dBm, and 10 dBm input power levels, demonstrating the device’s wideband performance and the close agreement between simulations and measurements.

Figure 9 shows the variation in V_out_ and PCE with frequency for the proposed rectifier in the 0.1–6 GHz frequency range at 0 dBm, 5 dBm, and 10 dBm input power levels. The measurement results indicate that PCE varies due to several uncontrollable factors such as diode modeling inaccuracies, manufacturing tolerances, environmental effects, soldering quality, signal generator instabilities, and coaxial cable losses. To clarify this variation, a quantitative assessment of the dominant sources of experimental uncertainty was conducted. The attenuation of the TA336 standard test lead with an SMA(m) coaxial cable used in the measurements is approximately 0.7 dB at 6 GHz, as specified in the manufacturer’s datasheet [21]. This value was independently verified using an HP8566B spectrum analyzer (Agilent Technologies, Santa Clara, CA, USA) and compensated for prior to the experiments. As a result, the cable loss was not treated as a systematic reduction in the calculated PCE, but rather as a limited residual uncertainty remaining after compensation. The RF signal generator employed in the measurements has an output power accuracy on the order of ±0.9 dB, which introduces a relative uncertainty in the PCE calculation depending on the applied input power level [22]. In addition, the manufacturer’s datasheet indicates that the dielectric constant of the FR4 substrate varies between 4.3 and 4.6 with frequency, leading to frequency-dependent variations in the measured PCE [23].

Figure 9a shows the variation in V_out_, which is used in the PCE calculation, as a function of frequency. The calculated PCE values are given in Figure 9b, and it is seen that at an input power of 10 dBm and a frequency of 2.9 GHz, the maximum PCE is 72.8% in simulations and 76.4% in measurements. Additionally, the measurement results show that a PCE above 60% is observed in the frequency ranges 1.8–3.3 GHz and 4.1–4.5 GHz at 10 dBm input power. The measurements also demonstrate that PCE remains above 50% over the wide bandwidth from 1.6 GHz to 5.1 GHz at 10 dBm input power.

Furthermore, the measurement results indicate that the proposed rectifier maintains its wideband performance at 0 dBm and 5 dBm input power levels, with PCE values between 30% and 50%. It is also observed that the rectifier operates efficiently within the 915 MHz–5.8 GHz operating range of the HSMS-2860 Schottky diodes, while the PCE significantly decreases outside this range.

Table 4 summarizes the performance parameters of the proposed UWB rectifier and compares them with other recent studies in the literature. The obtained data confirm that the study is consistent with the literature and that all performance criteria were considered when compared with other studies. According to Table 4, the proposed UWB rectifier operates over a wide frequency range with a fractional bandwidth of 105%, based on the center frequency. This enables power transfer across a broad frequency spectrum and minimizes frequency-shift issues during transmission. Furthermore, the proposed rectifier operates over a wide input power range of 15 dBm and exhibits strong tolerance to input power fluctuations. Therefore, the study demonstrates wideband operation in both frequency and input power domains. In addition, with its 19 × 27 mm^2^ compact size, the proposed design is smaller than many similar works in the literature and can be integrated into systems more easily. Another advantage is that the design is fabricated on FR4, a cost-effective substrate despite being more lossy than commonly used high-performance substrates in the literature.

## 5. Conclusions

In this study, a UltraWideBand microwave rectifier suitable for WPT systems and capable of efficient operation in the sub-6 GHz 5G bands was designed, analyzed, and experimentally validated. During the design process, a four-parallel shunt-diode topology was proposed to compensate for the variations in diode impedance with frequency and input power. This approach significantly improved impedance stability compared to single- or dual-diode structures reported in the literature.

Simulation and experimental results demonstrated that the rectifier consistently achieved PCE values exceeding 50% in the 1.6–5.1 GHz frequency range, covering the sub-6 GHz 5G bands. Moreover, the rectifier maintained PCE values above 50% over a wide input power range (1 dBm to 16 dBm), confirming its capability to operate efficiently under variable power conditions. Despite using a lossy substrate such as FR4, achieving a maximum PCE of 76% at 10 dBm and 2.9 GHz is quite remarkable. The compact dimensions of the rectifier and its fabrication on a low-cost FR4 substrate also facilitate its integration into practical WPT applications. When evaluated in terms of overall performance parameters, comparisons with the literature show that this work is either superior or comparable to other studies in the same category.

Furthermore, the study demonstrated that the high-frequency operation of the proposed rectifier is limited by the parasitic behavior of the Schottky diodes and the frequency-dependent variations in the dielectric constant and loss tangent of the FR4 substrate. These effects become increasingly pronounced as the upper edge of the operating band is approached. In future work, the high-frequency performance can be further improved by employing lower-loss substrates and diode technologies with reduced parasitic effects, enabling rectifiers with a wider operating range.

In conclusion, the proposed UWB rectifier provides an effective solution for IoT, WSN, RFID, and 5G-based WPT applications thanks to its high efficiency across a wide frequency band, its strong tolerance to varying input power levels, its compact structure, and its low-cost fabrication advantages.

## Figures and Tables

**Table 1:** Fundamental electrical and parasitic parameters of the HSMS-286x Schottky diode series used in the impedance calculations of the proposed rectifier.

*I_S_* (A)	*R_s_* (Ω)	*C_j_*_0_ (pF)	*V_j_* (V)	*n*	*m*	*C_p_* (pF)	*L_s_* (nH)
5 × 10^−8^	6.0	0.18	0.65	1.08	0.5	0.08	2

**Table 2:** Dimensions of the optimized transmission-line sections used in the proposed rectifier, including the width and length parameters for each line to achieve wideband impedance matching.

Name	Width (mm)	Length (mm)	Name	Width (mm)	Length (mm)
TL1	1.13	2	TL10	1.4	2.61
TL2	1.25	4.03	TL11	0.15	10
TL3	1.2	1	TL12	0.15	3.90
TL4	1.2	1	TL13	0.15	3.90
TL5	1.25	3.61	TL14	0.15	1
TL6	1.2	2	TL15	0.84	2.76
TL7	0.19	5.65	TL16	0.15	1
TL8	0.32	5	TL	0.2	2
TL9	0.51	1.83			

**Table 3:** Dimensions of the interconnecting structural elements between transmission-line sections.

Name	W1 (mm)	W2 (mm)	W3 (mm)	W4 (mm)
CROSS1	0.63	1.25	0.63	1.25
CROSS2	0.63	1.25	0.63	1.25
TEE1	1.13	1.2	1.25	
TEE2	1.2	1.2	1.25	
TEE	0.2	0.2	0.2	

**Table 4:** Performance comparison of the proposed ultra-wideband rectifier with recent state-of-the-art wideband rectifier designs, including bandwidth, efficiency, input power dynamic range, maximum PCE, substrate type, and physical size.

Ref	Year	Bandwidth (GHz) PCE > 50%	Dynamic Range (dBm) PCE > 50%	Max. PCE	Size (mm^2^)	Substrate
[10]	2024	3–5.8 @18 dBm	12–22 * @ 4 GHz	%66 @4 GHz @18 dBm	12 × 6	NA
[24]	2024	0.1–3.2 * @18 dBm	4–22 * @0.9 GHz	%80 @0.9 GHz @19 dBm	19 × 18	F4B
[17]	2024	1.3–3.8 * @7 dBm	(−3)–16 * @2 GHz	%82 @2.2 GHz @13 dBm	14 × 15	Taconic TLY-5
[25]	2025	1.3–3.3 @5 dBm	(−5)–13 * 2.8 GHz	%71 @2.8 GHz 8 dBm	19 × 69	NA
[26]	2025	1.4–2.5 @8 dBm	(−5)–13 1.6 GHz	%72 @1.6 GHz 8 dBm	33 × 36	RO4003
This Work	2025	1.6–5.1 @10 dBm	1–16 @3 GHz	%76 @2.9 GHz @10 dBm	19 × 27	FR4

* Estimated from graphs NA: Not Available.

## Data Availability

The raw data supporting the conclusions of this article will be made available by the author on request.

## Abbreviations

The following abbreviations are used in this manuscript:

WPT	Wireless Power Transfer
IoT	Internet of Things
RFID	Radio Frequency Identification
WSNs	Wireless Sensor Networks
DC	Direct Current
UWB	Ultra-wide Band
